# Discovery of Novel NMR-Based Biomarkers and Interpretable Machine Learning Models for Risk Prediction of Rheumatoid Arthritis

**DOI:** 10.3390/metabo16030153

**Published:** 2026-02-25

**Authors:** Hong Lin, Rui Wang, Linyan Lu, Ping Tian, Xiaodi Yang, Lianbo Xiao, Qing-Hua Li, Guo-Qiang Lin

**Affiliations:** 1 State Key Laboratory of Discovery and Utilization of Functional Components in Traditional Chinese Medicine, Innovation Research Institute of Traditional Chinese Medicine, Shanghai University of Traditional Chinese Medicine, 1200 Cailun Road, Shanghai 201203, China; 22023650@shutcm.edu.cn (H.L.); 22024764@shutcm.edu.cn (L.L.); tianping@shutcm.edu.cn (P.T.); lingq@sioc.ac.cn (G.-Q.L.); 2 Department of Orthopedic Surgery, Guanghua Hospital Affiliated to Shanghai University of Traditional Chinese Medicine, Shanghai 200052, China; wr1997tcm@163.com; 3 Guanghua Clinical Medical College, Guanghua Hospital Affiliated to Shanghai University of Traditional Chinese Medicine, Shanghai 200052, China

**Keywords:** NMR-based biomarkers, metabolite, lipoprotein subfractions, rheumatoid arthritis, risk prediction

## Abstract

**Background:** Early diagnosis of rheumatoid arthritis (RA) remains challenging due to the limited performance of existing serum biomarkers. This exploratory study aimed to identify novel serum metabolite and lipoprotein biomarkers for RA and to develop interpretable machine learning models for screening. **Methods**: This study employed ^1^H-NMR metabolomics to analyze serum from 77 RA patients and 70 healthy controls, quantifying 38 endogenous metabolites and 112 lipoprotein parameters. Seven key biomarkers were identified using multiple criteria and Least Absolute Shrinkage and Selection Operator (LASSO) regression. The dataset was split into training and testing sets (7:3 ratio), and four machine learning models were constructed. The Random Forest (RF) model was further interpreted using the SHapley Additive exPlanations (SHAP) method. **Results**: The selected biomarkers, including formic acid and High-density lipoprotein 4 phospholipids (H4PL), showed significant associations with RA. In the internal test set, the RF model demonstrated promising discriminatory ability. Additionally, a proof-of-concept regression model for predicting the Disease Activity Score in 28 joints (DAS-28) score was developed, explaining a portion of its variance (R^2^ = 0.548) in this cohort. **Conclusions**: This exploratory, single-center study identifies a novel panel of potential biomarkers for RA and provides a preliminary, interpretable predictive tool. The findings, particularly the internally validated high performance of certain markers, are hypothesis-generating and underscore the need for validation in larger, multi-center cohorts. The DAS-28 prediction model also warrants further investigation.

## 1. Introduction

RA is a chronic autoimmune disease characterized by persistent synovitis, progressive joint destruction, and extra-articular manifestations involving multiple organ systems [1]. Patients with rheumatoid arthritis are at increased risk for cardiovascular disease and experience increased mortality if it occurs [2]. The global prevalence of RA is approximately 1%, with women affected about three times more frequently than men, and the disease is associated with high disability rates [3,4]. Early diagnosis is therefore critical for improving patient outcomes.

Currently, RA diagnosis relies primarily on clinical evaluation (e.g., swollen and tender joint counts), imaging modalities such as X-ray and ultrasound for assessing joint damage [5,6], and serological biomarkers, including rheumatoid factor and anti-cyclic citrullinated peptide (anti-CCP) antibodies [7,8,9]. However, these diagnostic approaches have substantial limitations [10]. RF lacks specificity, as it can also be detected in other autoimmune conditions such as sjogren’s syndrome and systemic lupus erythematosus [11,12,13]. Moreover, up to 40% of RA patients test negative for anti-CCP antibodies [14,15], and the reliance on these serological markers reduces diagnostic sensitivity in seronegative RA cases [16]. Disease activity scores, such as DAS-28, provide a rapid and low-cost assessment tool, but they are prone to misclassifying disease activity and to cases of “pseudo-remission” [17,18]. In addition, conventional X-ray imaging is insufficiently sensitive to detect early lesions, limiting its clinical utility for early diagnosis [19].

These limitations underscore the urgent need to identify novel biomarkers that can enhance diagnostic accuracy and address unmet clinical needs. In this study, we employed high-resolution ^1^H-600 MHz NMR spectroscopy combined with multivariate statistical analysis to perform a comprehensive metabolomic analysis of serum samples from RA patients and healthy controls.

NMR can be applied to the analysis of various biological fluids, including blood, urine, cerebrospinal fluid, and synovial fluid [20]. It offers several advantages, such as being noninvasive, highly reproducible, nondestructive, and requiring simple sample preparation and recovery [21]. In addition to providing comprehensive information on lipoprotein subclasses, NMR simultaneously enables quantitative measurement of metabolite concentrations [22]. This approach captures alterations in the overall metabolic network and allows early disease warning, a capability that conventional clinical methods lack. The IVDr platform utilizes standardized pulse sequences, including Nuclear Overhauser Enhancement Spectroscopy (NOESY) and Carr–Purcell–Meiboom–Gill (CPMG), for data acquisition. By integrating algorithms such as Bayesian Automated Metabolite Analyzer (BATMAN) and Bayesian Spectroscopy Integration Library (BAYESIL) with the Electronic Reference To access In vivo Concentrations (ERETIC) quantitative method, it enables automated metabolite identification and accurate absolute quantification [23]. Its key strength lies in a fully standardized and automated workflow, providing high-throughput and highly reproducible analytical capabilities essential for clinical translation. We therefore aimed to leverage this high-precision analytical technique to identify metabolites and lipoprotein profiles that undergo significant changes in RA patients, and to screen for novel and reliable RA biomarkers. These potential biomarkers may be involved in key pathophysiological processes, including dyslipidemia, inflammatory responses, energy metabolism, oxidative stress, and gut microbiota–host co-metabolism. Such findings are expected to provide new scientific insights into RA pathogenesis, improve diagnostic efficiency, and inform precision therapeutic strategies.

## 2. Materials and Methods

### 2.1. Study Design and Participants

**Figure 1:**
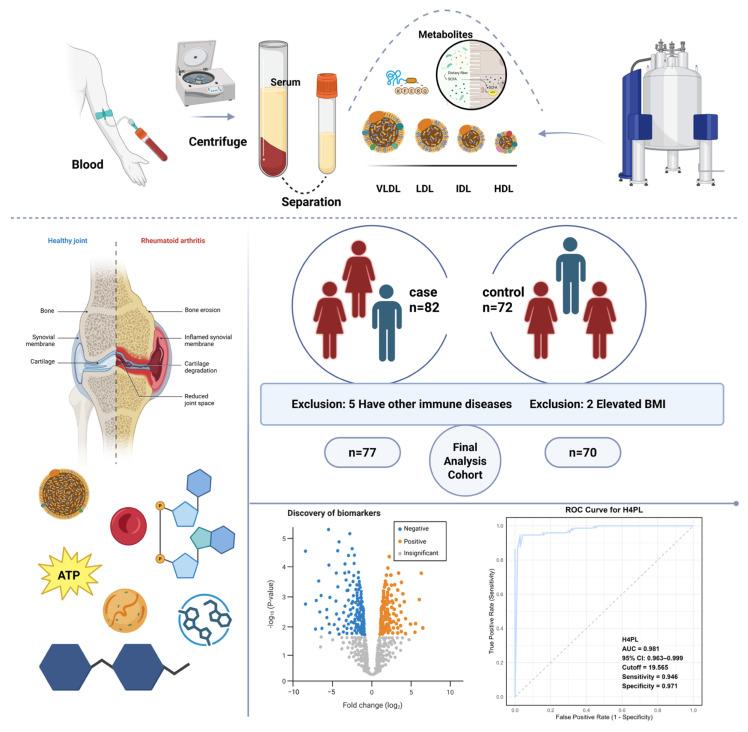
Serum metabolomics workflow for RA based on NMR spectroscopy. This schematic was created with a full license from BioRender.com.

This prospective, single-center study used data from an independent clinical trial cohort (ChiCTR2500096245), which was conducted at Guanghua Hospital Affiliated to Shanghai University of Traditional Chinese Medicine and initiated in December 2024. The study aimed to identify serum biomarkers for RA using nuclear magnetic resonance (NMR) metabolomics. A total of 82 RA patients and 72 age- and sex-matched healthy controls were enrolled. RA diagnosis adhered to the 2010 ACR/EULAR classification criteria. Key exclusion criteria included severe cardiac, hepatic, renal or cerebral comorbidities; active or chronic infections; other systemic rheumatic diseases; cortico-steroid use within the last six months; and malignancy. To minimize pre-analytical variability, all blood samples were collected in the morning (between 8:00 and 11:00 a.m.) after an overnight fast. The study protocol was approved by the hospital ethics committee (approval no. 2024-K-76), and written informed consent was obtained from all participants. Sample selection for analysis was performed based on pre-defined criteria, as shown in Figure 1. From the initially enrolled 154 participants, which included 82 RA patients and 72 healthy controls, seven individuals were excluded prior to metabolomics and machine learning analysis. Specifically, five RA patients were excluded due to comorbid systemic autoimmune diseases such as systemic lupus erythematosus or Sjögren’s syndrome, as these conditions could confound the RA-specific metabolic profile. Additionally, two healthy controls were excluded because their body mass index exceeded 30 kg/m^2^, a threshold considered to represent a significant metabolic abnormality. Consequently, the final analyzed cohort comprised 77 RA patients and 70 healthy controls, totaling 147 participants.

### 2.2. Sample Processing and NMR Metabolomics

Fasting serum samples were stored at −80 °C. For analysis, the samples were thawed and mixed with NMR plasma buffer (Bruker Switzerland AG, Zürich, Switzerland) before being transferred to 5 mm NMR tubes (Bruker Switzerland AG, Zürich, Switzerland). ^1^H-NMR spectra were acquired at 310 K on a Bruker 600 MHz spectrometer. Quantitative metabolic and lipoprotein profiling was performed using the Bruker IVDr Lipoprotein Subclass Analysis (B.I.-LISA) platform. This yielded absolute concentrations for 38 endogenous metabolites and 112 parameters covering 15 lipoprotein subfractions (Table S1, Supporting Information), including particle concentrations and lipid/apolipoprotein contents (e.g., cholesterol, triglycerides, Apo-A1 and Apo-B100) [24].

### 2.3. Data Preprocessing

Variables with >30% missing values were excluded. Any remaining missing data were imputed using multiple imputation (IBM SPSS Statistics 26.0). The distribution of continuous variables was assessed using the Shapiro–Wilk test. All variables were subsequently standardized via Z-score normalization (mean = 0, SD = 1) to ensure comparability for modeling.

### 2.4. Feature Screening and Selection

Differential features between RA patients and healthy controls were identified through a two-step process. First, an Orthogonal Projections to Latent Structures-Discriminant Analysis (OPLS-DA) model was constructed using R v4.4.3. Features with a Variable Importance in Projection (VIP) score greater than 1 were considered discriminatory. Secondly, univariate significance was assessed using Wilcoxon tests with false discovery rate (FDR) correction (adjusted *p* < 0.05). Features that fulfilled both criteria (VIP > 1 and FDR < 0.05) were retained.

Subsequently, LASSO regression was applied to select variables and reduce multicollinearity. Prior to analysis, all continuous predictor variables were standardized (Z-score normalization, achieving a mean of 0 and a standard deviation of 1). The initially screened features were included, with RA status as the response variable. To ensure reproducibility, a random seed was set (seed = 1234). The optimal penalty parameter (λ) was determined via five-fold cross-validation (without repetition). Specifically, we applied the one-standard-error (λ_(1SE)) rule to select the most parsimonious and stable model, yielding an optimal λ value of 0.00021. This process identified seven non-zero coefficient variables as key predictors. The LASSO regression analysis was performed using the “glmnet” package (version 4.1-8) in R.

### 2.5. Machine Learning Model Development for RA Classification

The cohort was randomly split into a training set (70%) and an internal validation set (30%). Using the selected predictors, four classification algorithms were trained: Logistic Regression (LR), RF, Support Vector Machine (SVM) and eXtreme Gradient Boosting (XGBoost). Hyperparameters were optimized via an initial grid search, followed by manual fine-tuning. Model training employed five-fold cross-validation on the training set. The final model’s performance was evaluated on the held-out validation set using the area under the ROC curve (AUC), accuracy, recall, and F1 score.

### 2.6. Predictive Modeling for Disease Activity and Clinical Tool Development

A separate random forest regression model was developed to predict RA disease activity, as quantified by the DAS-28 score, using serum metabolic and lipoprotein profiles. Model performance was evaluated using the coefficient of determination (R^2^) and root mean square error (RMSE). Diagnostic plots (actual versus predicted values, learning curves and residual plots) were generated to evaluate the model’s fit and the distribution of errors.

To evaluate the clinical utility of the model for stratifying disease activity, we converted the continuous scores predicted by the regression model into three categories based on internationally accepted DAS-28 clinical cut-offs: <2.6 for “Remission”, 2.6–5.2 for “Low to Moderate Activity”, and >5.2 for “High Disease Activity”. The classification accuracy for each group was then calculated by comparing the model-predicted category against the clinical category derived from the actual DAS-28 score. It is important to note that due to the limited sample size, the classification accuracy for the “High Disease Activity” group (*n* = 4) should be interpreted with caution and is not statistically reliable.

To facilitate clinical translation, a nomogram was constructed to predict RA risk. Candidate biomarkers were first screened via univariable logistic regression. Significant variables (*p* < 0.05) were then incorporated into a final multivariable logistic regression model, which formed the basis of the nomogram. Model performance was internally validated using bootstrap resampling (1000 iterations). Discrimination was assessed by the C-statistic (AUC), and calibration was evaluated via bootstrap-corrected calibration curves (1000 resamples) with calibration intercept, slope, and mean absolute error (MAE). Owing to the complete separation of formic acid (non-overlapping values between cases and controls), the ridge regression penalty (penalty = 20) was applied to shrink predicted probabilities to a plausible range (0.004–0.972) and obtain stable coefficient estimates.

### 2.7. Pathway Analysis

KEGG pathway enrichment analysis of the differential metabolites identified by NMR was performed using MetaboAnalyst v6.0 to identify perturbed metabolic pathways in RA.

## 3. Results

### 3.1. Study Workflow and Participants’ Demographic Characteristics

Workflow and Demographic Characteristics of Participants. The overall study workflow and participant recruitment strategy are illustrated in Figure 1. A total of 82 RA patients and 72 healthy individuals were enrolled from Shanghai Guanghua Integrated Traditional Chinese and Western Medicine Hospital between January and May 2025. To minimize potential confounding, all blood samples were collected during a fixed morning time window (8:00–11:00 a.m.). Furthermore, RA patients were enrolled under strict inclusion and exclusion criteria (as detailed in the Section 2) to control for major comorbidities and medication use. Baseline clinical characteristics indicated that the two groups had comparable body mass index (BMI) and sex distribution; the median BMI values were 22.41 and 22.60, respectively, and the proportion of females was higher in both groups (Table S2, Supporting Information).

### 3.2. Serum Metabolite and Lipoprotein Variations

**Figure 2:**
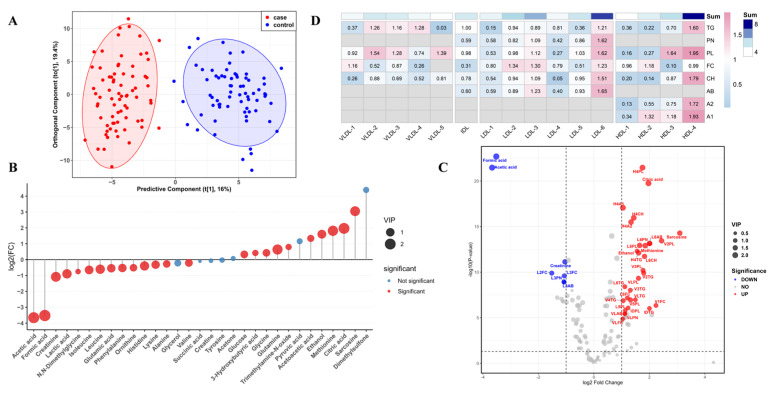
Analysis of Serum NMR-based Metabolomics and Lipoprotein Profiling. (**A**) Orthogonal partial least squares-discriminant analysis (OPLS-DA). This analysis was applied to identify differential metabolites and lipoproteins between groups. (**B**) Differential analysis of serum metabolites. Metabolites differing between the RA patient and healthy control cohorts were screened using multiple criteria. (**C**) Volcano plot. This plot is used for the rapid identification of lipoprotein subfractions and metabolites that exhibit pronounced alterations along with statistical significance. The dashed lines indicate the thresholds: the vertical dashed line on the x-axis represents a VIP value greater than 1, while the horizontal dashed line on the y-axis represents an absolute log_2_(FDR-adjusted *p*-value) greater than 0.585. (**D**) Variable importance of lipoprotein subfractions. The “Sum” represents the weighted sum of each individual component that constitutes the lipoprotein subfraction particles.

Serum Metabolite Distribution and Alterations. NMR-based metabolomics analysis of serum samples from 77 RA patients and 70 healthy controls was performed using the Bruker IVDr platform. A total of 38 serum metabolites and 112 lipoprotein subclasses were relatively quantified, and their features were comprehensively analyzed. The OPLS-DA model demonstrated a clear separation between groups (Figure 2A) with strong explanatory and predictive power. Upon rigorous validation (permutation test, *n* = 200), the model showed a high explained variance (R^2^Y = 0.882) and a robust cross-validated predictive ability (Q^2^ = 0.849), both of which were statistically significant (*p* = 0.005, CV-ANOVA; Supplementary Figure S2A). Combined with univariate analysis (Mann–Whitney U test, q < 0.05), amino acid and glucose-derived metabolites were screened (Figure 2B). Seven metabolites (VIP > 1, q < 0.05, FC > 1.5) were identified as candidate biomarkers (Figure S1B, Supporting Information). Among these, ethanol, methionine, citric acid, and sarcosine were significantly elevated in the RA group, while acetic acid, formic acid, and creatinine were significantly decreased. Pathway enrichment analysis (Figure S1C, Supporting Information) revealed that energy metabolism was significantly enriched, suggesting marked dysregulation of metabolic energy processes in RA. Conversely, the amino acid metabolic pathway exhibited a low ratio and lack of statistical significance, indicating a relatively minor contribution.

HDL and LDL Subclasses as Potential Risk Factors for RA. In terms of lipoprotein profiling, ^1^H-NMR identified 112 lipoprotein-related parameters (Table S3, Supporting Information). Distinct alterations were observed between RA patients and healthy controls (Figure 2C), with notable changes in VLDL, LDL, and HDL subclasses. Further subgroup analysis (Figure 2D) revealed that structural components of HDL-4 (TG, CH, PL, Apo-A1, Apo-A2) and LDL-6 (TG, CH, FC, PL, Apo-B100) exhibited significant VIP values (VIP > 1), suggesting their close association with RA and potential as risk biomarkers.

To further explore the role of LDL-6 and HDL-4 in RA pathogenesis, correlations were analyzed between their main structural components (TG, CH, FC, PL, Apo-A1, Apo-A2, and Apo-B100) and serum metabolites (Figure S2, Supporting Information). Both LDL-6 and HDL-4 components showed significant correlations with carbohydrate metabolism: positive correlations with citric acid and glutamine, and negative correlations with formic acid and acetic acid. Regarding amino acid metabolism, HDL-4 was positively correlated with methionine and sarcosine, while LDL-6 was positively correlated with sarcosine. Although limited literature has reported on the involvement of LDL and HDL subclasses in carbohydrate and amino acid metabolism, our findings suggest that LDL-6 and HDL-4 may influence these pathways directly or indirectly, thereby contributing to RA progression. The underlying mechanisms warrant further investigation.

### 3.3. Lasso Regression for Feature Selection in RA

**Figure 3:**
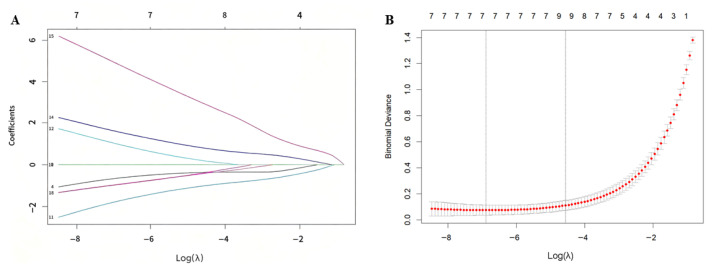
Results of the LASSO regression. (**A**) Selection process for the regularization parameter λ. (**B**) Coefficient paths of the 17 variables across different values of λ.

Variable selection and model construction were performed using the LASSO regression, which automatically selects relevant features and simplifies the model while effectively handling multicollinearity. The initial model included 17 features derived from 7 metabolites and 2 lipoprotein subfractions (LDL-6 and HDL-4). Ultimately, 7 variables with non-zero coefficients were retained as significant predictors (H4PL, Low-density lipoprotein 6 free cholesterol (L6FC), Citric acid, Creatinine, Ethanol, Formic acid, and Acetic acid) (Figure 3).

While the remaining 10 variables were identified as redundant and shrunk to zero. This simplified model structure enhances the interpretability of the biomarkers and strengthens the reliability and clinical relevance of the findings.

### 3.4. Evaluation of Individual Variables for Predicting RA

The filtered biomarkers demonstrate distinct expression patterns between the patient and control groups (Figure S3A, Supporting Information). The concentration distributions of the seven LASSO-selected biomarkers are visually compared in Supplementary Figure S4. Their concentrations show strong contrasts across groups, suggesting a potentially important association with RA. These biomarkers represent promising candidates for further investigation. Evaluation of Individual Predictive Variables. To assess the diagnostic performance of the seven variables selected by LASSO regression (H4PL, L6FC, citric acid, creatinine, ethanol, formic acid, and acetic acid), ROC curve analyses were performed for each variable. Several diagnostic performance metrics, including AUC, 95% CI, optimal cutoff values, sensitivity, and specificity, were calculated (Table 1). These results indicated that each variable exhibited good predictive potential when used individually, with citric acid, H4PL, formic acid, and acetic acid showing particularly strong discriminative ability (AUC > 0.95). Notably, formic acid achieved near-perfect discrimination (AUC ≈ 1.000) in this cohort, which is visually supported by the complete separation of its concentration distribution between groups (Figure S4, Supporting Information). This provided a solid foundation for constructing multivariable diagnostic models.

The clinical relevance of the seven biomarkers was further evaluated by examining their correlations with RA disease activity, as measured by DAS-28 [25,26]. Spearman’s rank correlation analysis revealed no significant associations. While our cohort analysis showed no significant correlation between them (Figure S3B, Supporting Information), the identified biomarkers could potentially serve as diagnostic markers to differentiate patients from healthy individuals.

### 3.5. Machine Learning for RA Risk Prediction

Machine Learning Models for Predicting RA Risk. To systematically evaluate the combined diagnostic value of the selected features, four commonly used machine learning algorithms were applied to construct RA classification models: RF, Support Vector Machine (SVM), EXtreme Gradient Boosting (XGBoost), and Logistic Regression (LR). Model performance was comprehensively compared using accuracy, precision, recall, F1-score, and AUC.

As summarized in Table S4 (Supporting Information), RF and XGBoost achieved superior performance, with RF demonstrating greater robustness and generalizability. Therefore, RF was selected as the optimal model for further analysis. Confusion matrices and five-fold cross-validation for both training and test sets are shown in Figure S5 (Supporting Information), confirming that overfitting was avoided. As summarized in Table S4 (Supporting Information), both RF and XGBoost achieved superior performance, with the RF model demonstrating greater robustness and generalizability. On the independent test set, the RF model exhibited perfect discriminative ability with an AUC of 1.000 (95% CI: 1.000–1.000). To further assess model reliability, we analyzed the calibration curve Figure S5A (Supporting Information), which showed good agreement between predicted probabilities and observed frequencies (Hosmer-Lemeshow test: χ^2^ = 1.20, *p* = 0.752). The probability distribution plot Figure S5B (Supporting Information) further revealed complete separation between the predicted probability distributions of RA patients and healthy controls, indicating perfect discriminative capacity on the current dataset. Based on its comprehensive superior performance, RF was selected as the optimal model for further analysis. Confusion matrices and five-fold cross-validation results for both training and test sets are shown in Figure S5C,D (Supporting Information), confirming the absence of overfitting.

**Figure 4:**
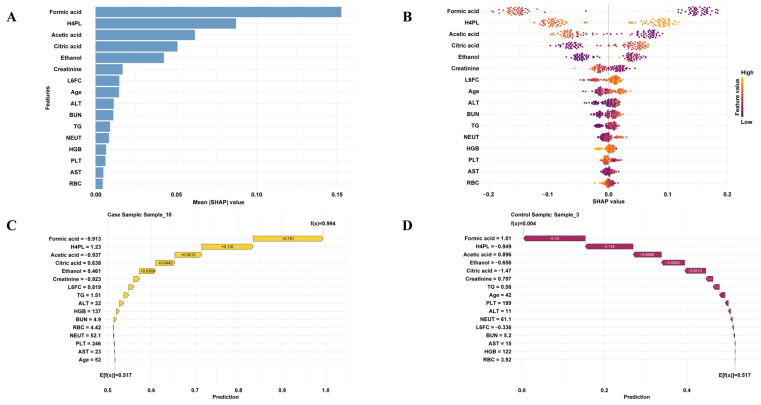
Model interpretation using the SHAP method. (**A**) The bar plot ranks the mean absolute impact of features on model predictions using SHAP values. (**B**) Beeswarm plot. Each point represents the SHAP value for an individual sample, with orange color indicating a higher feature value. (**C**) Waterfall plot illustrating the contribution of each feature’s SHAP value to the final prediction for a representative RA patient. (**D**) Waterfall plot showing the contribution of each feature’s SHAP value to the final prediction for a representative healthy control.

To interpret the predictive contributions of individual biomarkers, SHAP analysis was applied to the RF model. This method enabled both global and local interpretability: global analysis identified the overall importance of each feature, while local analysis highlighted feature contributions to predictions for individual patients. As shown in (Figure 4A,B), SHAP summary plots ranked features by their mean absolute SHAP values, revealing that the top four predictors associated with increased RA risk were formic acid, H4PL, acetic acid, and citric acid. Local-level interpretations further illustrated how specific biomarker profiles contributed to predictions in individual patients. This interpretability provides actionable insights for clinical decision-making and supports personalized and precision-oriented RA risk assessment.

As shown in (Figure 4C,D), the predicted probabilities of RA for the patient and healthy control were 99.4% and 0.4%, respectively. The SHAP dependence plots provide further insight into how individual features influence the model output. (Figure S6A–G, Supporting Information) illustrates the relationship between the actual values of these seven features and their SHAP values. A SHAP value greater than zero indicates that the feature contributes positively to the RA prediction. The partial dependence plot for formic acid reveals that as its concentration increases, the SHAP value decreases. This implies a reduced predicted risk of RA, suggesting that formic acid acts as a “protective factor”. Similarly, elevated levels of acetic acid are associated with negative SHAP values. In contrast, H4PL and citric acid are identified as risk factors for RA. Increased levels of these metabolites are linked to positive SHAP values, significantly enhancing the model’s positive prediction. Furthermore, the interaction plots in Figure S6H,I (Supporting Information) demonstrate significant interactions between formic acid and citric acid, as well as between formic acid and acetic acid. When formic acid is at a low level, high citric acid or low acetic acid exerts a positive effect on the model’s prediction of RA.

### 3.6. Predicting RA Risk with Random Forest and Multivariable Logistic Regression

**Figure 5:**
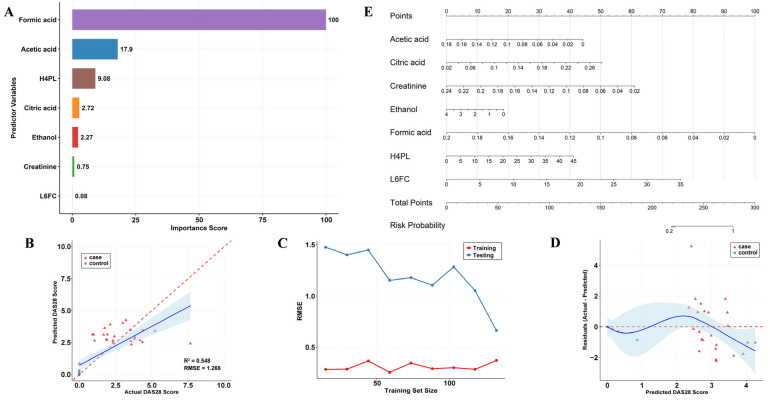
Model Development and evaluation of the RA prediction model. (**A**) Importance ranking of the predictor variables in the model. (**B**) Scatter plot of model-predicted values versus actual observations. (**C**) Learning curve of the model for assessing potential overfitting. (**D**) Residual distribution plot of the model. (**E**) Nomogram for individualized prediction of RA risk.

We then employed a random forest regression algorithm to develop a prediction model for the RA DAS-28, based on serum metabolite and lipoprotein profiles. Formic acid and acetic acid emerged as the two most important variables for predicting DAS-28, followed by H4PL and citric acid. Other variables, along with interaction terms, also contributed partially to the predictive ability (Figure 5A).

The model demonstrated robust predictive performance (Figure 5B), with a coefficient of determination (R^2^) of 0.548 and a root mean square error (RMSE) of 1.266. These results indicate that the model explains more than half of the variance in DAS-28 scores, underscoring its clinical relevance. The actual versus predicted values were distributed closely along the diagonal, though some scattered points were observed, suggesting consistent predictive trends across the entire score range. The learning curve (Figure 5C) confirmed that the model did not overfit and exhibited good generalization ability. Residual analysis (Figure 5D) showed that the prediction errors approximately followed a normal distribution without systematic bias, further validating the model’s reliability. To assess the model’s ability to stratify disease activity, we converted the predicted continuous DAS-28 scores into categories using clinical cut-offs (Remission: <2.6; Low-to-Moderate: 2.6–5.2; High: >5.2). The model demonstrated good clinical utility in identifying patients in “Remission” (*n* = 33, accuracy 72.7%) and those with “Low-to-Moderate Activity” (*n* = 40, accuracy 80.0%). However, the model failed to correctly classify patients in the “High Disease Activity” group, which had a very small sample size (*n* = 4, accuracy 0.0%). This result primarily reflects the limitation of an underpowered subgroup analysis rather than an intrinsic failure of the model. Relevant data are provided in Table 2. The model effectively distinguished between the healthy control and patient groups. This indicates that the model is largely unbiased and maintains good discriminative ability. Finally, to predict RA disease risk. A predictive nomogram was constructed based on these seven biomarkers using multivariable logistic regression, providing an intuitive tool for quantifying individual risk of RA development. (Figure 5E). The penalized logistic regression model demonstrated near-perfect discrimination (AUC = 0.9998, bootstrap-corrected AUC = 0.9998) with a Nagelkerke R^2^ of 0.7889. Calibration analysis revealed excellent model fit: calibration intercept = 0.005 (ideal = 0), calibration slope = 0.996 (ideal = 1), and mean absolute error = 0.015 (Supplementary Figure S7). Predicted probabilities ranged from 0.004 to 0.972, with no extreme values. These findings confirm that, despite complete separation of formic acid, the strongly penalized model achieves both outstanding discriminative ability and reliable absolute risk estimation in the current dataset.

## 4. Discussion

RA is a chronic systemic autoimmune disease characterized by symmetrical polyarthritis of small joints in the hands and feet [27,28]. Persistent inflammation promotes the development of cardiovascular complications such as atherosclerosis [29]. RA itself is considered an independent risk factor for cardiovascular disease [30], with overall mortality in RA patients being about 50% higher than that of the general population, mainly due to cardiovascular complications [31,32].

In this study, we performed a preliminary analysis of lipoprotein subclass alterations in RA and identified lipoprotein particles with strong predictive value. We found that H4PL and L6FC have potential as novel biomarkers for RA monitoring, which has rarely been reported. Phospholipids, as crucial precursors for inflammatory mediators, participate in and promote the sustained progression of inflammation. Membrane phospholipids, particularly phosphatidylcholine, are hydrolyzed by phospholipase A2 (PLA2) [33,34]. In the synovium of RA patients, PLA2 activity is significantly elevated, leading to the massive release of arachidonic acid from membrane phospholipids. This arachidonic acid is then metabolized via the cyclooxygenase and lipoxygenase pathways into various potent pro-inflammatory mediators, such as prostaglandins and leukotrienes [35,36]. HDL generally exerts anti-inflammatory, antioxidant, and reverse cholesterol transport functions and is regarded as a “good” lipoprotein [37,38,39]. However, in chronic inflammatory environments, the proteomic and lipidomic composition of HDL undergoes remodeling, losing its protective functions and turning into pro-inflammatory HDL [40,41]. In RA, free cholesterol forms crystals that serve as an endogenous danger signal for the NLRP3 inflammasome. This cascade triggers the maturation and release of powerful pro-inflammatory cytokines, including IL-1β, directly amplifying inflammation within the joint [42,43]. LDL transports cholesterol from the liver to peripheral tissues [44]. In our study, nearly all HDL-4 subclass particles were significantly elevated. Increases in Apo-A1, Apo-A2, and PL suggest not only numerical expansion but also particle enlargement, while CH elevation results from higher PL. The abnormal rise in TG indicates disrupted lipid exchange, as HDL normally contains more cholesterol and relatively low TG [45].

Our results further indicate marked disturbances in endogenous metabolites in RA patients. Specifically, acetic acid, formic acid, and creatinine were decreased, whereas ethanol and citric acid were increased. These changes collectively point to metabolic reprogramming, mitochondrial dysfunction, enhanced oxidative stress, and potential gut microbiota dysbiosis, processes tightly associated with chronic inflammation in RA.

Acetic acid and formic acid are important SCFAs. As the most abundant SCFA in the gut, acetic acid plays key roles in mitigating inflammation and maintaining intestinal barrier integrity [46,47]. A decrease in its level may impair the regulation of the immune system, thereby exacerbating systemic inflammatory responses [48]. We observed that the levels of formic acid and acetic acid in RA patients were below the cutoff value, while their concentrations were significantly higher in healthy controls. The presence of endogenous ethanol in RA patients, as detected in serum, may stem from gut microbiota fermentation or anaerobic metabolism due to local tissue hypoxia [49,50]. This implicates both gut dysbiosis and a hypoxic synovial microenvironment in altering ethanol levels, phenomena that are indicative of broader systemic metabolic disturbances in RA.

Creatinine reduction is mainly associated with RA-related muscle wasting and cachexia, driven by chronically elevated inflammatory cytokines such as TNF-α and IL-6, which accelerate protein catabolism and muscle loss [51,52]. This finding directly confirms RA as a consumptive disease and aligns with symptoms of fatigue and weakness in patients.

Citric acid is a key metabolite at the entry point of the TCA cycle. Previous studies have reported decreased citric acid in urine and synovial fluid, often linked to the Warburg effect in immune cells [53,54]. Interestingly, in our study, citric acid was elevated in serum from RA patients. This discrepancy may result from sample type differences, reflecting variations between local microenvironments and systemic metabolism. We speculate that increased serum citrate indicates global metabolic reprogramming in RA patients. To meet the energy demands of chronic inflammation and lipid biosynthesis for immune cell proliferation, enhanced glycolysis and TCA flux may lead to citrate accumulation in serum.

A direct, intra-cohort comparison with RF/anti-CCP was precluded by the absence of these measurements, a study limitation. To contextualize our findings, we compare our panel’s performance with published meta-analyses: anti-CCP shows pooled sensitivity 61.7–71%, specificity 95–97.1% (AUC ≈ 0.95); RF shows sensitivity 69–77%, specificity 73–85% (AUC ≈ 0.82); anti-MCV shows sensitivity 68.6–71%, specificity 89–94.2% (AUC ≈ 0.89) [55,56]. In routine cohorts, anti-CCP2/3 assays yield sensitivities of 76.9–80.9% and specificities of 61.0–69.5% [9]. Our metabolite/lipoprotein panel achieved promising accuracy (AUC = 1.000) in internal validation, comparable to or exceeding these conventional markers. Importantly, our panel reflects distinct metabolic perturbations (energy metabolism, oxidative stress, gut–joint axis) rather than autoantibody-mediated immunity, suggesting complementary diagnostic value, particularly for seronegative or early RA. These indirect comparisons require caution due to population and methodological differences. External validation and prospective head-to-head studies are urgently needed to establish additive clinical utility.

Despite these novel findings, this study should be regarded as exploratory in nature, and several limitations warrant consideration. First, the overall sample size is relatively modest (*n* = 147), and critically, the high disease activity subgroup consisted of only four patients, resulting in extremely low and statistically unreliable classification accuracy in this subgroup. Although we employed LASSO regularization and five-fold cross-validation to mitigate overfitting, the limited sample size may still restrict the generalizability of our model to broader populations, and statistical power may be insufficient to detect more subtle yet clinically relevant features. Therefore, these preliminary findings require rigorous external validation in independent cohorts.

Furthermore, limitations related to missing data handling warrant consideration. Multiple imputation (predictive mean matching, 20 imputations, 50 iterations) was applied to metabolites with missing values. However, the complete-case sample size was extremely small (*n* = 6 for cases, *n* = 7 for controls), leading to substantial sampling variability in the estimates of means and standard deviations. After imputation, the expanded sample size resulted in notable changes in the mean values of a few metabolites (e.g., 3-Hydroxybutyric acid and Ethanol in the control group). Sensitivity analyses confirmed that the majority of metabolites remained stable before and after imputation, but the observed shifts in these specific variables warrant cautious interpretation. Moreover, multiple imputation relies on the missing at random assumption; if data were missing not at random, the imputed results could be biased. RF model achieved perfect discriminative performance on the independent test set (AUC = 1.000). While this may reflect genuinely strong biological separation conferred by the selected metabolites and lipoprotein subfractions, the possibility of overfitting or chance findings due to limited sample size cannot be entirely excluded. Although we employed cross-validation, learning curve analysis, and a held-out test set to minimize overfitting, as an exploratory study, independent external validation in larger cohorts remains a necessary prerequisite to confirm the model’s generalizability. Notably, this study adopted a single-center design, which is inherently susceptible to selection bias and unmeasured confounding. Although metabolite concentrations were normalized and sensitivity analyses confirmed the robustness of the imputation procedure, potential batch effects or technical variability may still have influenced some metabolite measurements.

Although the seven identified biomarkers demonstrated excellent performance in internal validation, their clinical utility and mechanistic relevance require further investigation. The findings of this study should be considered hypothesis-generating, providing directions for subsequent research. Future work will involve multicenter collaborations to collect larger and more diverse datasets, which are essential for robust validation and eventual clinical translation of our model.

## 5. Conclusions

In conclusion, this exploratory study utilized NMR-based metabolomics to profile serum from a single-center cohort of RA patients and healthy controls. We identified several novel lipoprotein subclasses (e.g., H4PL, L6FC) and metabolites (e.g., formic acid, citrate) that are significantly altered in RA, implicating potential disruptions in energy metabolism, oxidative stress, and the gut–joint axis. Machine learning models incorporating these biomarkers showed promising discriminatory ability in our internal validation, though the optimal performance observed requires cautious interpretation and confirmation in independent, external cohorts. Importantly, we provide a preliminary, interpretable framework for RA screening and, for the first time, a metabolomics-based model that correlates with disease activity (DAS-28), explaining a substantial proportion of its variance in our dataset. Collectively, our findings offer novel, hypothesis-generating insights into the metabolic landscape of RA and present candidate biomarkers for future development. The immediate clinical translation is limited by the study’s sample size and single-center design; thus, the primary contribution lies in laying a groundwork for subsequent validation and mechanistic investigation. Ongoing studies are focused on external validation and deeper functional analysis of the identified pathways.

## Figures and Tables

**Table 1:** Performance of Serum Metabolites and Biomarkers for Disease Detection.

Variable	Test AUC (95%CI)	Train Cut-Off	Test Sensitivity	Test Specificity
Acetic acid	0.967 (0.921–1.000)	0.035 mmol/L	0.870	0.952
Citric acid	0.945 (0.879–1.000)	0.128 mmol/L	0.870	0.905
Creatinine	0.755 (0.603–0.906)	0.089 mmol/L	79.7	77.1
Formic acid	1.000 (0.999–1.000)	0.045 mmol/L	1.000	0.952
Ethanol	0.865 (0.732–0.999)	0.207 mmol/L	0.826	1.000
H4PL	0.985 (0.956–1.000)	19.450 mg/dL	0.957	1.000
L6FC	0.659 (0.494–0.825)	4.540 mg/dL	0.696	0.714

Note: Cutoff values were determined in the train set and performance was evaluated in an independent test set. Values are rounded to three decimal places. The AUC (with 95% CI in parentheses) represents the overall diagnostic ability of each biomarker. The optimal cut-off value was determined using the Youden index. Sensitivity and specificity percentages correspond to the performance at the specified cut-off value. The exceptionally high AUC values, particularly for formic acid, are reflective of the pronounced separation in biomarker levels observed in this cohort (Figure S4).

**Table 2:** Prediction accuracy of the regression model for RA disease activity.

DAS-28 Score Range	Disease Activity	Prediction Accuracy %
<2.6	Remission	72.7
2.6–5.2	Low to Moderate Disease Activity	80.0
>5.2	High Disease Activity	0.0

## Data Availability

The source code, including differential computation, feature selection, prediction models, and plotting, was publicly available on GitHub: https://github.com/qdlqh2001/NMR-Based-Biomarkers-and-Interpretable-Machine-Learning-Models.git (accessed on 13 February 2026). The data that support the findings of this study are available from the corresponding author upon reasonable request. Restrictions apply to the availability of these data, which are not publicly available due to privacy or ethical considerations.

## Supplementary Materials

The following supporting information can be downloaded at: https://www.mdpi.com/article/10.3390/metabo16030153/s1. Table S1: List of density range of lipoprotein subfractions; Table S2: Clinical and demographic characteristics of the participants.; Figure S1: OPLS-DA Model Validation and Identification of Discriminatory Metabolites; Table S3: Overview of Respective Lipoprotein Particle Composition; Figure S2: Correlation between Abnormal Lipoprotein Subfraction Particles and Metabolites; Figure S3: Biomarkers are evaluated comprehensively from all aspects; Figure S4: Distribution of the seven serum biomarkers selected by LASSO regression Table S4: Machine Learning Model Metrics; Figure S5: Performance evaluation of the Random Forest model; Figure S6. SHAP Dependency Plots; Figure S7. Bootstrap-corrected calibration curve of the penalized logistic regression model.

## Abbreviations

RA	Rheumatoid Arthritis
NMR	Nuclear Magnetic Resonance
NOESY	Nuclear Overhauser Enhancement Spectroscopy
CPMG	Carr-Purcell-Meiboom-Gill
ERETIC	Electronic Reference To access In vivo Concentrations
BATMAN	Bayesian Automated Metabolite Analyzer
BAYESIL	Bayesian Spectroscopy Integration Library
^1^H-NMR	Proton Nuclear Magnetic Resonance
LASSO	Least Absolute Shrinkage and Selection Operator
RF	Random Forest
SHAP	SHapley Additive exPlanations
AUC	Area Under the Curve
OPLS-DA	Orthogonal Partial Least Squares-Discriminant Analysis
VIP	Variable Importance in Projection
FC	Fold Change
HDL	High-Density Lipoprotein
LDL	Low-Density Lipoprotein
VLDL	Very Low-Density Lipoprotein
TG	Triglycerides
CH	Cholesterol
PL	Phospholipids
Apo	Apolipoprotein
ROC	Receiver Operating Characteristic
CI	Confidence Interval
SVM	Support Vector Machine
XGBoost	eXtreme Gradient Boosting
DAS-28	Disease Activity Score in 28 joints
RMSE	Root Mean Square Error
SCFAs	Short-Chain Fatty Acids
TCA	Tricarboxylic Acid Cycle
KEGG	Kyoto Encyclopedia of Genes and Genomes
PCA	Principal Component Analysis
FDR	False Discovery Rate
MSE	Mean Squared Error
LR	Logistic Regression
H4PL	High-density lipoprotein 4 phospholipids
L6FC	Low-density lipoprotein 6 free cholesterol
