# Supplementary material for: Discovery of Novel NMR-Based Biomarkers and Interpretable Machine Learning Models for Risk Prediction of Rheumatoid Arthritis
Source: Metabolites. 2026 Feb 25;16(3):153. doi: 10.3390/metabo16030153 (PMC13028240; doi:10.3390/metabo16030153)
Supplement: Supplementary file 1 [file metabolites-16-00153-s001.zip › metabolites-4139628-supplementary.pdf]

metabolites

Table S1. List of density range of lipoprotein subfractions.

Major Class	Density Range (kg/L)	Subclass	Density Range (kg/L)
VLDL	0.950 - 1.006		
IDL	1.006 - 1.019		
LDL	1.019 - 1.063	LDL-1	1.019 - 1.031
		LDL-2	1.031 - 1.034
		LDL-3	1.034 - 1.037
		LDL-4	1.037 - 1.040
		LDL-5	1.040 - 1.044
		LDL-6	1.044 - 1.063
HDL	1.063 - 1.210	HDL-1	1.063 - 1.100
		HDL-2	1.100 - 1.112
		HDL-3	1.112 - 1.125
		HDL-4	1.125 - 1.210

Table S2. Clinical and demographic characteristics of the participants.

Characteristic	case(n=77)	control(n=70)	P value
Age	63.00 (53.25,68.00)	47.00 (38.00,55.00)	<0.001
BMI	22.41 (20.45,24.00)	22.60 (21.12,25.50)	0.210
WBC	5.72 (4.54,7.00)	5.45 (4.80,6.60)	0.742
RBC	4.16 (3.87,4.40)	4.36 (4.14,4.66)	<0.001
HGB	126.00 (116.00,134.00)	132.50 (122.25,145.00)	<0.001
PLT	188.50 (156.00,227.50)	219.50 (191.25,258.75)	0.004
NEUT%	59.70 (55.02,72.08)	56.90 (48.17,60.98)	<0.001
ALT	21.50 (14.00,32.75)	10.50 (8.00,16.00)	<0.001
AST	26.00 (21.00,32.75)	23.79 (19.00,24.97)	0.001
ALP	70.00 (52.25,84.75)	70.54 (65.25,74.42)	0.817
BUN	5.50 (4.73,6.60)	4.60 (4.10,5.38)	<0.001
UA	291.00 (247.98,320.90)	292.00 (242.00,358.75)	0.374
GGT	20.00 (15.00,36.00)	27.11 (15.25,29.01)	0.713
CREA	61.10 (51.30,70.88)	61.00 (54.00,72.00)	0.424
TC	5.00 (4.77,5.21)	4.75 (4.36,5.51)	0.118
TG	1.44 (1.29,1.58)	1.12 (0.82,1.45)	<0.001
HDL	1.50 (1.42,1.58)	1.47 (1.21,1.69)	0.373
LDL	3.19 (2.92,3.34)	3.18 (2.82,3.70)	0.240
DAS-28	2.97 (2.03, 3.80)		
sex			0.243
male	17（22.08）	20（28.57）	
female	60（77.92）	50（71.43）	

WBC, White Blood Cell count; **RBC**, Red Blood Cell count; **HGB**, Hemoglobin; **PLT**, Patelets; **NEUT**, Neutrophil Percentage; **ALT**, Alanine Aminotransferase; **AST**, Aspartate Aminotransferase; **ALP**, Alkaline Phosphatase; **BUN**, Blood Urea Nitrogen; **UA**, Uric Acid; **GGT**, γ-Glutamyl Transferase; **Cr**, Creatinine; **TC**, Total Cholesterol; **TG**, Triglycerides.

The Mann-Whitney U test results are presented as medians, and the Chi-squared test results are presented using percentiles.

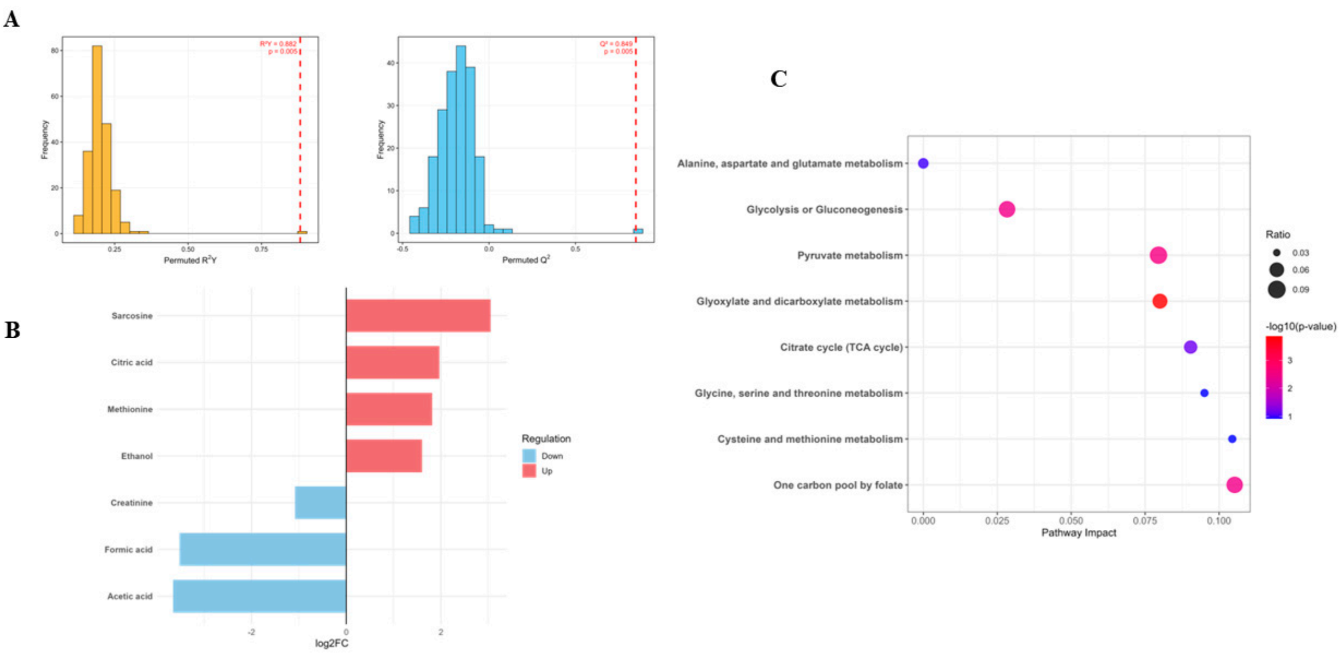

Figure S1. OPLS-DA Model Validation and Identification of Discriminatory Metabolites. A: Permutation test validating the OPLS-DA model. B: Identification of signature metabolites with VIP > 1 and q < 0.05. C: Enriched Metabolic Pathways.

Table S3. Overview of Respective Lipoprotein Particle Composition.

	TG	CH	FC	PL	Apo-A1	Apo-A2	Apo-B100	PN
VLDL	✓	✓	✓	✓	—	—	✓	✓
IDL	✓	✓	✓	✓	—	—	✓	✓
LDL	✓	✓	✓	✓	—	—	✓	✓
HDL	✓	✓	✓	✓	✓	✓	—	—
VLDL-1	✓	✓	✓	✓	—	—	—	—
VLDL-2	✓	✓	✓	✓	—	—	—	—
VLDL-3	✓	✓	✓	✓	—	—	—	—
VLDL-4	✓	✓	✓	✓	—	—	—	—
VLDL-5	✓	✓	✓	✓	—	—	—	—
LDL-1	✓	✓	✓	✓	—	—	✓	✓
LDL-2	✓	✓	✓	✓	—	—	✓	✓
LDL-3	✓	✓	✓	✓	—	—	✓	✓
LDL-4	✓	✓	✓	✓	—	—	✓	✓
LDL-5	✓	✓	✓	✓	—	—	✓	✓
LDL-6	✓	✓	✓	✓	—	—	✓	✓
HDL-1	✓	✓	✓	✓	✓	✓	—	—
HDL-2	✓	✓	✓	✓	✓	✓	—	—
HDL-3	✓	✓	✓	✓	✓	✓	—	—
HDL-4	✓	✓	✓	✓	✓	✓	—	—

TG, tryglicerides; **CH**, Cholesterol; **FC**, Free Cholesterol; **PL**, Phospholipids; **Apo**, Apolipoprotein; **PN**, Particle numbers; **✓**, detected and quantified. **—**, not detected or not quantified. Lipoprotein subclasses are numbered based on their density, with higher numbers corresponding to greater density.

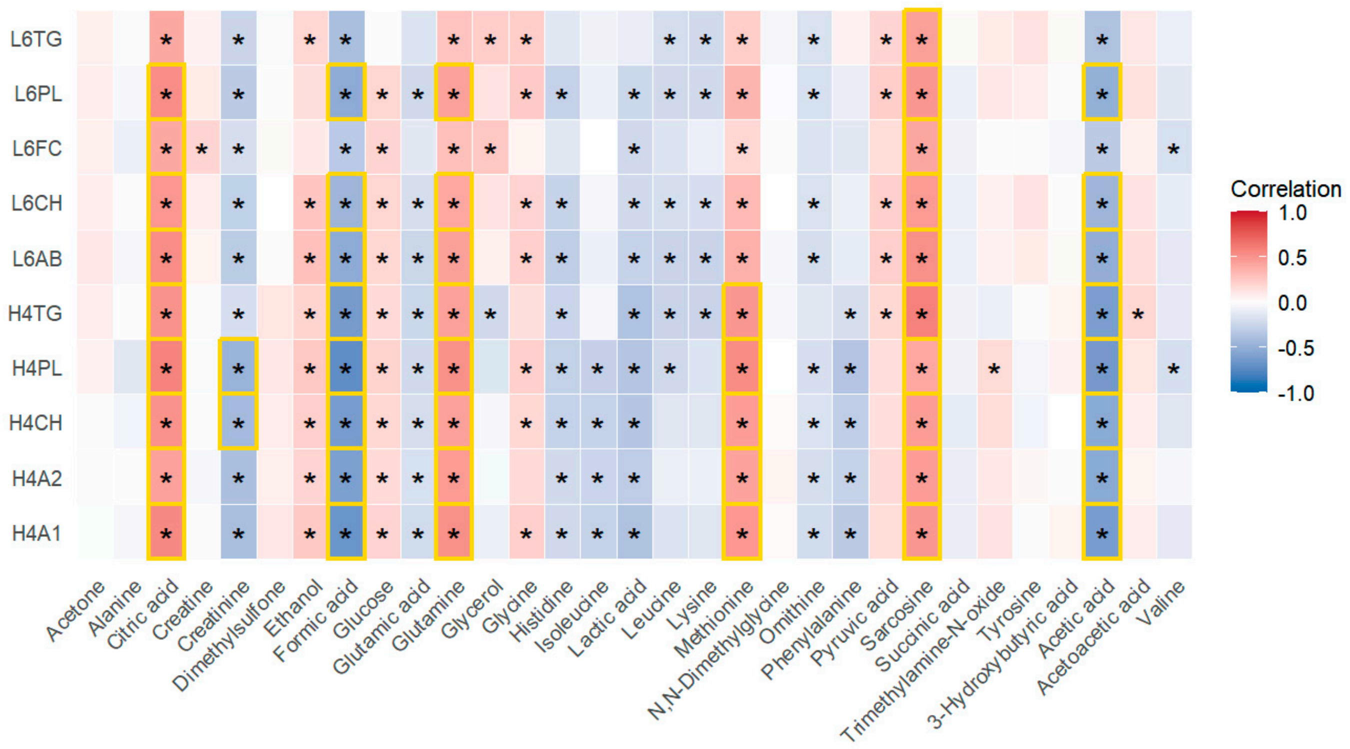

Figure S2. Correlation between Abnormal Lipoprotein Subfraction Particles and Metabolites. Correlation analysis of LDL-6 and HDL-4 levels with plasma Endogenous Metabolites.

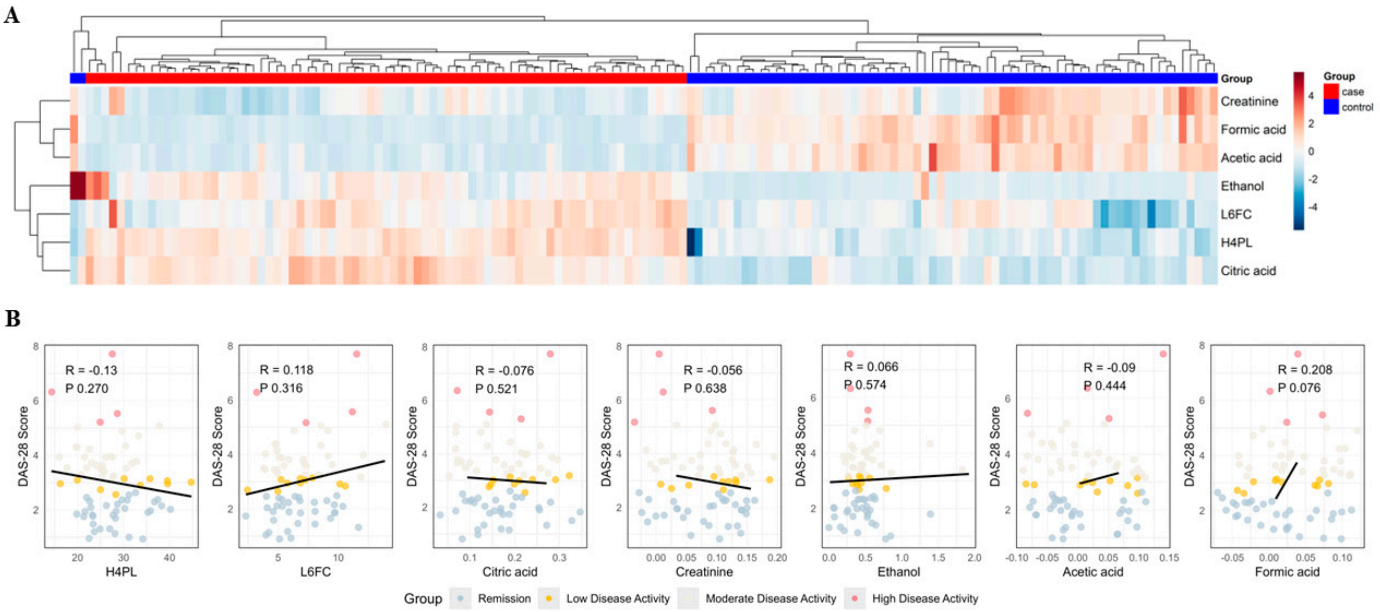

Figure S3. Biomarkers are evaluated comprehensively from all aspects. A: The heatmap illustrates the differences between groups. B: DAS28 Score Range: remission (< 2.6), low (2.6 to 3.2), moderate (3.2 to 5.1), or high (> 5.1).

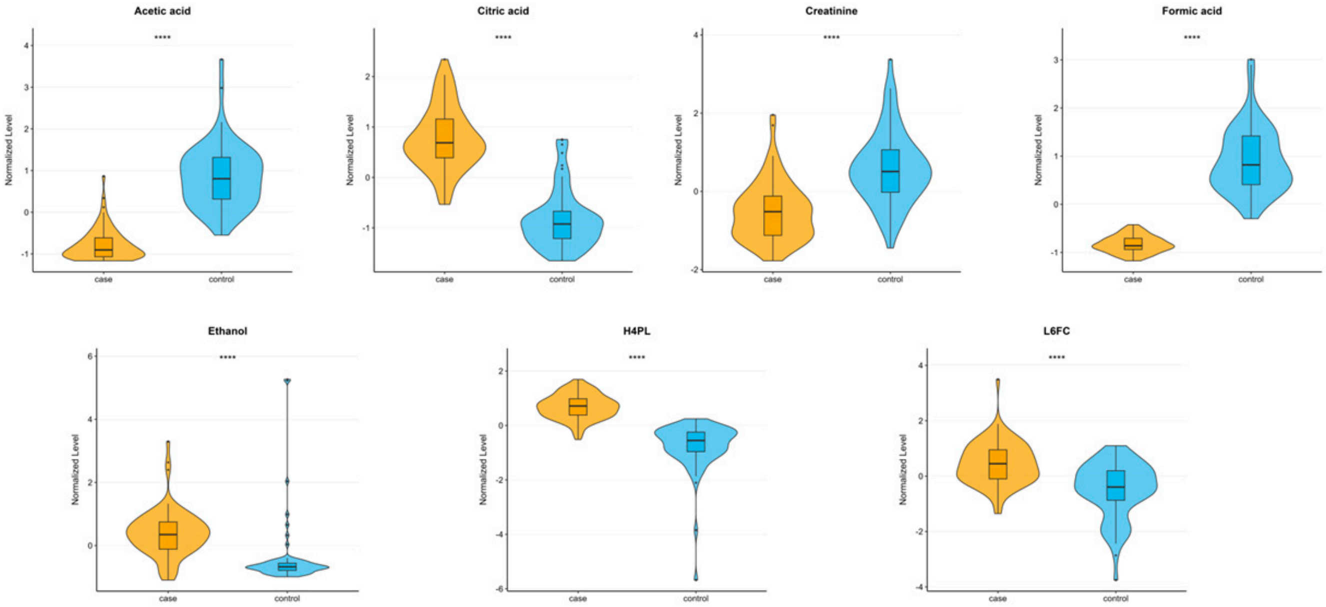

Figure S4. Distribution of the seven serum biomarkers selected by LASSO regression. Each plot shows the data density (shape) and the interquartile range (box), with a horizontal line inside the box indicating the median. Statistical significance was assessed using the Mann-Whitney U test (****$p < 0.0001$). Notably, formic acid shows complete separation between groups, with the maximum value in the RA group being lower than the minimum value in the HC group, providing a visual explanation for its exceptionally high diagnostic performance (AUC $\approx$ 1.000) in this cohort. The pronounced distribution differences for other biomarkers (e.g., acetic acid, H4PL, citric acid) also underpin their high individual AUC values (Table 1).

Table S4. Machine Learning Model Metrics.

Model	AUC	Accuracy	F1	Precision	Recall
RF	1.000	1.000	1.000	1.000	1.000
SVM	1.000	0.978	0.979	1.000	0.958
XGboost	1.000	1.000	1.000	1.000	1.000
LR	1.000	0.955	0.955	1.000	0.913

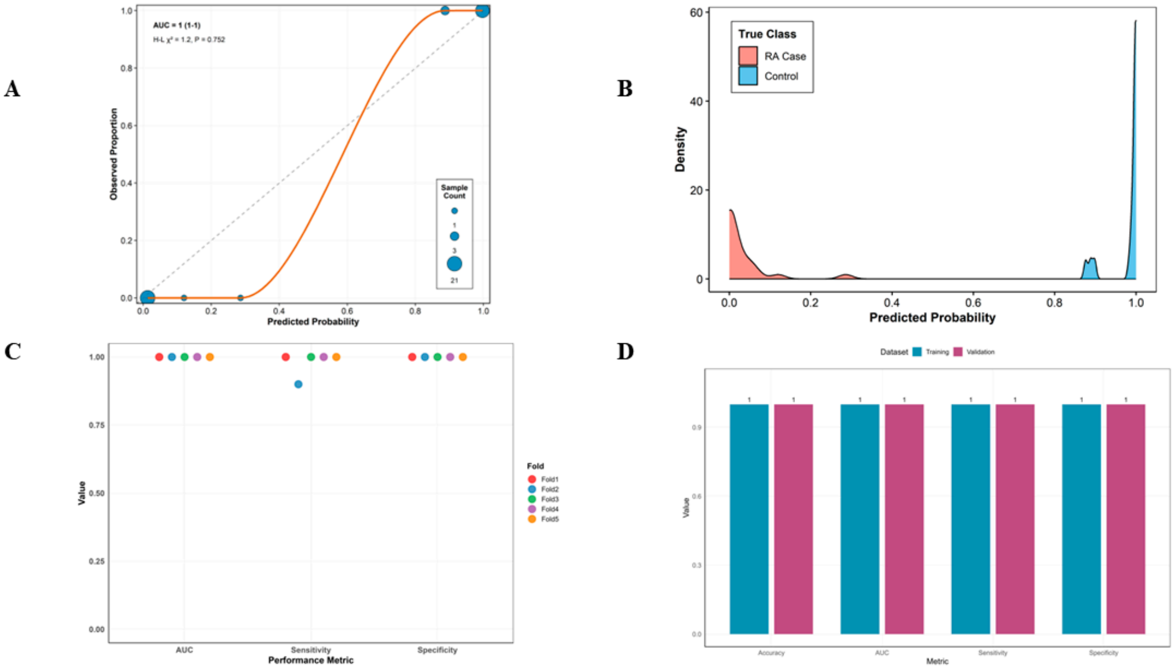

Figure S5. Performance evaluation of the Random Forest model. A: Calibration curve of the Random Forest model on the test set. B: Probability distribution of predicted risk scores for RA cases and healthy controls on the test set, illustrating complete separation between the two groups. C: Five-fold cross-validation on the training set. D: Confusion matrix on the training set.

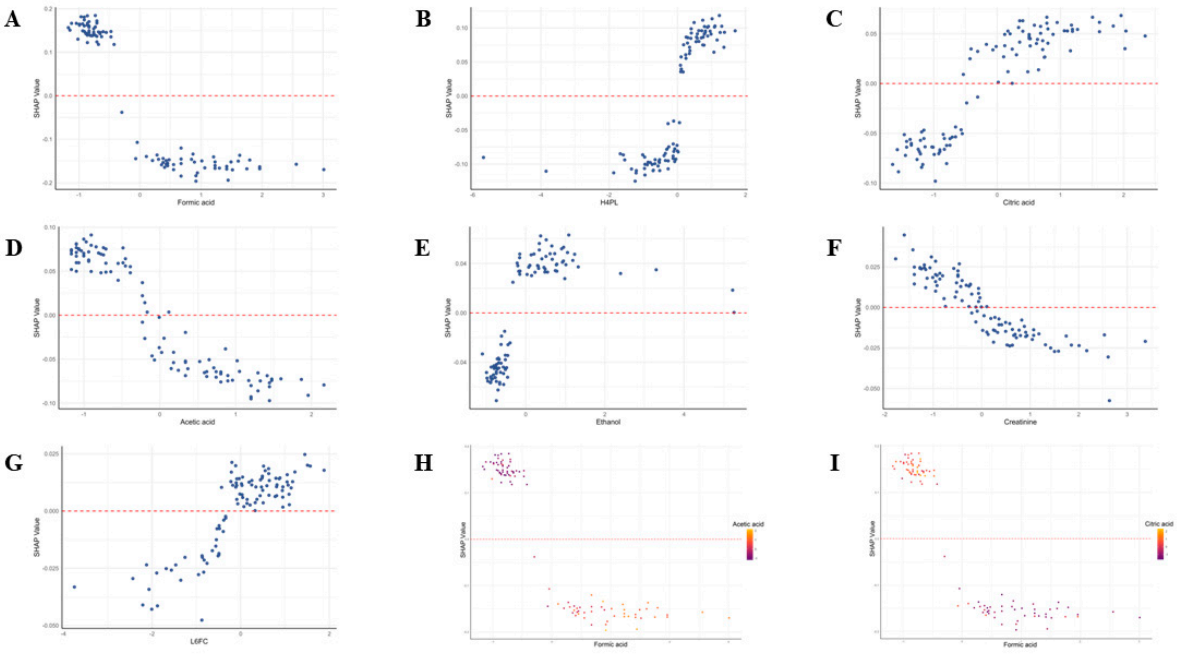

Figure S6. SHAP Dependency Plots. A single-feature dependency plot illustrates how one individual feature influences the model's predictions. A multi-feature dependency plot demonstrates how the interactions between features jointly impact the model's output.

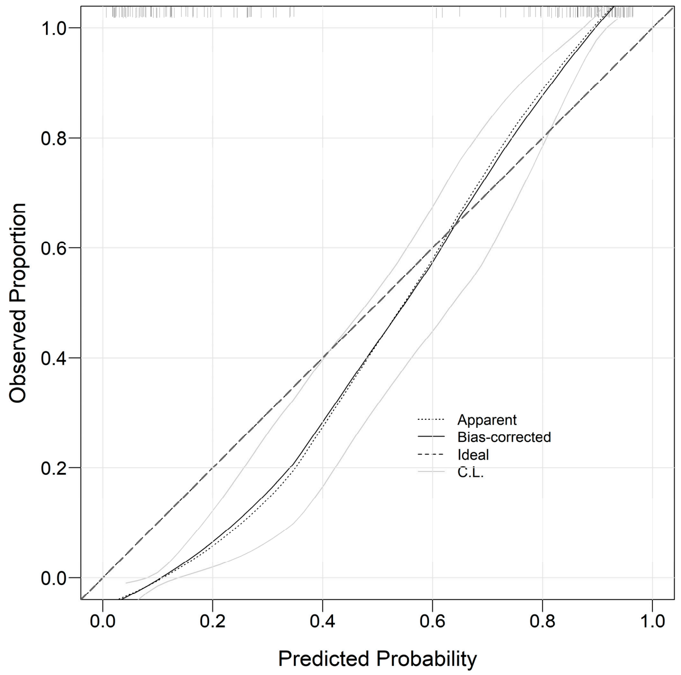

Figure S7. Bootstrap-corrected calibration curve of the penalized logistic regression model. The dashed diagonal line represents perfect calibration (intercept = 0, slope = 1). The solid black line indicates apparent calibration, and black circles with error bars show bias-corrected estimates with 95% confidence intervals (1,000 bootstrap resamples). After applying ridge penalty (penalty = 20), predicted probabilities were shrunk to the range of 0.004–0.972. The bias-corrected calibration intercept is 0.005 (95% CI: –0.012 to 0.022), the calibration slope is 0.996 (95% CI: 0.971–1.021), and the mean absolute error is 0.015, demonstrating excellent model calibration.
